# Co-prescription of metformin and glucagon-like peptide-1 receptor agonists and metformin-associated lactic acidosis: A case series 

**DOI:** 10.5414/CNCS111965

**Published:** 2026-05-18

**Authors:** Hamed Hussain, Mustafa Ali, Koravich Lorlowhakarn, Maria Christina Capistrano, Vaidyanathapuram S. Balakrishnan, Bertrand L. Jaber

**Affiliations:** 1 Department of Medicine,; 2 Division of Nephrology, Boston Medical Center – Brighton,; 3 Department of Medicine, Tufts University School of Medicine, and; 4 Department of Medicine, Boston University Chobanian & Avedisian School of Medicine, Boston, MA, USA

**Keywords:** metformin-associated lactic acidosis, metformin, GLP-1 receptor agonist, GIP and GLP-1 dual receptor agonist, drug-drug interaction, acute kidney injury, dialysis, renal replacement therapy

## Abstract

Metformin-associated lactic acidosis (MALA) is a rare phenomenon mostly described in the setting of impaired renal elimination or following an acute overdose. While metformin is first-line treatment for type 2 diabetes mellitus, glucagon-like peptide-1 (GLP-1) receptor agonists and glucose-dependent insulinotropic polypeptide (GIP) and GLP-1 dual receptor agonists are frequent add-on agents. Both classes of medications share common gastrointestinal adverse reactions, including decreased appetite, nausea, vomiting, and abdominal pain. We present a series of four critically ill patients with type 2 diabetes mellitus on high-dose metformin who presented with severe MALA following the introduction or dose escalation of a GLP-1 or GIP/GLP-1 dual receptor agonist, or during an acute gastrointestinal illness while also on a maintenance dose of a GLP-1 receptor agonist, requiring acute renal replacement therapy. Prudence must be exercised when co-prescribing these 2 classes of medications with close monitoring of kidney function, possible reduction in metformin dose, and emphasis on sick day rule teaching.

## Introduction 

Metformin-associated lactic acidosis (MALA) is a rare manifestation of metformin toxicity that occurs due to impaired renal elimination or following an acute overdose, and it is associated with a high case-fatality rate of 17 – 50% [1, 2]. The most reported gastrointestinal adverse reactions of metformin (≥ 30%) are diarrhea, nausea, flatulence, dyspepsia, vomiting, and abdominal pain, which are dose dependent and tend to be transient and reversible. Glucagon-like peptide-1 (GLP-1) receptor agonists and glucose-dependent insulinotropic polypeptide (GIP) and GLP-1 dual receptor agonists have become widely used in combination with metformin for the treatment of patients with type 2 diabetes mellitus. Similarly, these agents have common gastrointestinal adverse reactions (10 – 20%) [3], including decreased appetite, nausea, dyspepsia, abdominal pain, and constipation, and these tend to occur during dose escalation (i.e., rapid titration) and with higher doses. 

It has not been established whether the combined use of these two classes of medications has the potential to accentuate these gastrointestinal adverse reactions, which can result in more serious downstream clinical adverse events. We present 4 patients with type 2 diabetes mellitus on high-dose metformin who presented with severe MALA following the introduction or dose escalation of a GLP-1 or GIP/GLP-1 dual receptor agonist, or during an acute gastrointestinal illness while also on a maintenance dose of a GLP-1 receptor agonist, all of whom were successfully treated with renal replacement therapy. We review the current literature and provide prudent monitoring guidance when these medications are co-prescribed to minimize drug-drug interactions and potential serious adverse reactions. 

## Case reports 

### Case 1 

A 69-year-old woman with hypertension (on lisinopril), and poorly controlled type 2 diabetes mellitus (on metformin, linagliptin, glyburide, and semaglutide) presented to the hospital with nausea, vomiting, and decreased oral intake for 2 days. She was taking metformin immediate-release 1,000 mg twice daily, and 3 months earlier had been started on semaglutide 0.5 mg weekly. Two weeks prior to presentation, the dose was increased to 1.0 mg weekly, after which she started experiencing these symptoms. She was hypoglycemic (glucose of 41 mg/dL), which was corrected with oral glucose. However, she was severely lethargic requiring intubation. She had severe acute kidney failure with a blood urea nitrogen (BUN) of 79 mg/dL and a serum creatinine of 8.7 mg/dL (baseline of 1.1 mg/dL), severe hyperkalemia with a serum potassium of 7.9 mEq/L, severe high anion-gap metabolic acidosis with a serum bicarbonate of 4 mEq/L and a serum anion gap of 39 mmol/L, and severe hyperlactatemia with serum lactic acid level of 14.5 mmol/L. Arterial blood gas revealed a pH of 7.03. She was resuscitated with crystalloids and received intravenous insulin and calcium gluconate for hyperkalemia, and sodium bicarbonate infusion for her metabolic acidosis. She was anuric and blood pressure was labile requiring an infusion of norepinephrine and vasopressin. She received continuous veno-venous hemodialysis (CVVHD) for 65 hours for suspected MALA, resulting in resolution of her metabolic acidosis, followed by successful extubation and renal recovery. Serum metformin level was markedly elevated at 23 µg/mL. She was discharged to a short-term rehabilitation facility. At time of hospital discharge, metformin, linagliptin, semaglutide, and lisinopril were discontinued, and serum creatinine was 2.2 mg/dL. Post discharge, she was restarted on dulaglutide (but not metformin), which was well tolerated. 

### Case 2 

A 68-year-old man with longstanding type 2 diabetes mellitus (on metformin, tirzepatide (previously on dulaglutide), dapagliflozin, and insulin glargine), diabetic retinopathy, hypertension (on losartan and clonidine), hyperlipidemia (on atorvastatin), and nephrolithiasis presented to the hospital with 4 days of abdominal pain, nausea, vomiting, and decreased urine output. The patient was taking metformin immediate-release 1,000 mg twice daily and had been prescribed tirzepatide 5 mg weekly 5 months earlier, which is higher than the usual starting dose of 2.5 mg weekly. He had been previously evaluated for symptoms of abdominal pain and decreased oral intake but remained on tirzepatide and metformin. On presentation, he was found to be hypoglycemic (glucose of 31 mg/dL) treated with oral glucose and intravenous dextrose 10% in water. He was hypotensive and had severe acute kidney failure with a BUN of 60 mg/dL and a serum creatinine of 9.1 mg/dL (baseline of 0.7 mg/dL), severe hyperkalemia with a serum potassium of 7.8 mEq/L, high anion-gap metabolic acidosis with a serum bicarbonate of 15 mEq/L and a serum anion gap of 26 mmol/L, and hyperlactatemia with serum lactic acid level of 7.8 mmol/L. Arterial blood gas revealed a pH of 7.26. The urinalysis had trace ketones, and the urine sediment revealed coarse granular casts, consistent with acute tubular necrosis. A kidney ultrasound revealed no hydronephrosis. He received CVVHD for suspected MALA, which was discontinued after 7 hours due to persistent clotting of the extracorporeal circuit. Urine output improved without diuretic assistance, and with resolution of the metabolic acidosis and hyperkalemia. Serum metformin level was markedly elevated at 22 µg/mL. On hospital day 9, he was discharged home in stable condition. At time of hospital discharge, metformin, tirzepatide, and losartan were discontinued, and serum creatinine was 5.8 mg/dL, which improved to 1.9 mg/dL 10 weeks later. Post discharge, he was not restarted on a GIP/GLP-1 dual receptor agonist. 

### Case 3 

A 67-year-old man with type 2 diabetes mellitus (on metformin, semaglutide, and insulin glargine), hypertension (on lisinopril, hydrochlorothiazide, and amlodipine), hyperlipidemia (on atorvastatin), coronary artery disease status post coronary artery bypass surgery (on aspirin and prasugrel), and giant cell arteritis (on tocilizumab) presented to the hospital with chest pain for 1 day and abdominal pain, vomiting, and diarrhea for 5 days and was found to have mild hypoglycemia (glucose 50 – 60 mg/dL) requiring administration of dextrose 10% in water. The patient was on metformin immediate-release 1,000 mg in the morning and 1,500 mg in the evening and had been taking semaglutide 2.0 mg weekly for 2 years. He had severe acute kidney failure with a BUN of 77 mg/dL and a serum creatinine of 8.2 mg/dL (baseline value of 1.1 mg/dL), hyperkalemia with a serum potassium of 6.2 mEq/L, severe high anion-gap metabolic acidosis with a serum bicarbonate of 3 mmol/L, and a serum anion gap of 40 mmol/L, severe hyperlactatemia with a serum lactic acid of 20.2 mmol/L, and hyperketonemia with a serum beta-hydroxybutyrate level of 6.9 mmol/L, consistent with diabetic ketoacidosis. The venous pH was less than 6.8. Serial high-sensitivity cardiac troponin-T ruled out acute coronary ischemia. Computerized tomography scan of the abdomen and pelvis was unremarkable. The patient was intubated and placed on mechanical ventilation. He received an insulin infusion for diabetic keto-acidosis. For the severe acidemia, he received a sodium bicarbonate infusion, but blood pressure remained labile, and he required an infusion of norepinephrine and vasopressin. Due to severe metabolic acidosis and oliguria, he was initiated emergently on CVVHD for suspected MALA, which he required for 72 hours before his kidney function improved. He was eventually weaned off the vasopressors and was extubated. The serum metformin level was markedly elevated at 31 µg/mL. On hospital day 10, he was discharged to a short-term rehabilitation facility. At the time of hospital discharge, lisinopril, metformin, and semaglutide were discontinued, and serum creatinine was 1.4 mg/dL. Post discharge, he was not restarted on a GLP-1 receptor agonist. 

### Case 4 

A 67-year-old woman with a history of diabetes mellitus (on metformin, semaglutide, and insulin prot-aspart), hypertension (on lisinopril), hyperlipidemia (on rosuvastatin), anemia, and diverticulitis presented to the hospital with 1 week of nausea, vomiting, abdominal pain, and hypoglycemia. The patient was on metformin immediate-release 1,000 mg twice daily and had been taking semaglutide 2.0 mg weekly for 1.5 years. She had acute kidney failure with a BUN of 59 mg/dL and a serum creatinine of 5.3 mg/dL (unknown baseline, but no known history of chronic kidney disease), severe hyperkalemia with a serum potassium of 7.1 mEq/L, severe high anion-gap metabolic acidosis with a serum bicarbonate of 9 mEq/L and an anion gap of 40 mmol/L, and severe hyperlactatemia with a serum lactic acid level of 14.3 mmol/L. Arterial blood gas revealed a pH of 7.12. She was resuscitated with normal saline and initiated on a bicarbonate drip. The urine sediment revealed coarse granular casts, consistent with acute tubular necrosis. In the intensive care unit, she was hemodynamically stable but had worsening kidney failure, persistent hyperkalemia, and oliguria resistant to a loop diuretic trial. Due to suspicion for MALA, she underwent an emergent single hemodialysis session, and post dialysis, the metabolic acidosis corrected, and kidney function gradually improved. Serum metformin level was elevated at 8 µg/mL. On hospital day 4, she was discharged home in stable condition. At the time of hospital discharge, metformin, semaglutide, and lisinopril were discontinued, and serum creatinine was 3.0 mg/dL. Post discharge, she was not restarted on a GLP-1 receptor agonist. 

## Discussion 

Metformin and GLP-1 or GIP/GLP-1 receptor agonists are both widely used in the treatment of type 2 diabetes mellitus, in part because of their potential for cardiovascular risk reduction, with the indications for GLP-1 and GIP/GLP-1 receptor agonists expanding noticeably. The Food and Drug Administration (FDA)-approved indications for GLP-1 and GIP/GLP-1 receptor agonists primarily include type 2 diabetes, chronic weight management, cardiovascular risk reduction, obstructive sleep apnea, and metabolic dysfunction-associated steatohepatitis (MASH). It is not surprising that the prescription rate of semaglutide has risen rapidly, with an increase of 442% between January 2021 and December 2023 [4]. However, both classes of drugs have undesirable gastrointestinal adverse effects, mainly diarrhea with metformin (occurring in 30% or more), and decreased appetite, dyspepsia, abdominal pain, nausea, vomiting, and constipation with GLP-1 and GIP/GLP-1 dual receptor agonists (occurring in 10 – 20%) [3]. Although these agents are frequently used in combination, the possibility of gastrointestinal adverse effect synergism has not been systematically examined. 

Multiple mechanisms have been proposed for the gastrointestinal adverse reactions associated with the use of metformin, including alterations in organic cation transporter 1 (OCT1), direct serotoninergic-like effects, increased gut motility, direct or indirect increases in GLP-1, and disruptions in bile [5]. We can only speculate as to whether the co-prescription of a GLP-1 receptor agonist or a GIP/GLP-1 dual receptor agonist would potentiate the GLP-1 incretin-mediated mechanism, slowing down gastric emptying. Potential risk factors for metformin-induced gastrointestinal adverse reactions include rapid dose escalation, use of immediate-release formulations, chronic gastritis (e.g., *Helicobacter pylori* infection), and concomitant use of OCT1-inhibiting agents such as verapamil and proton pump inhibitors. Patients receiving the immediate-release formulation of metformin may be switched to the extended-release formulation, taken as once daily at the same total daily dose, up to 2,000 mg once daily, as gastrointestinal adverse reactions are less common with extended-release compared to immediate-release tablets, including less nausea and vomiting (7 vs. 26%) and less diarrhea (10 vs. 53%) [6]. On the other hand, the gastrointestinal adverse effects of GLP-1 and GIP/GLP-1 receptor agonists are dose-related and tend to occur during dose escalation and decrease over time. The exact mechanisms are not fully understood but include delayed gastric emptying or activation of centers involved in appetite regulation, satiety, and nausea [7]. One patient (case 1) was co-prescribed linagliptin, a dipeptidyl peptidase-4 (DPP-4) inhibitor, which might have potentiated the gastrointestinal side effects of the GLP-1 receptor agonist, resulting in volume depletion and acute kidney injury. Although DPP-4 inhibitors have not been associated with an increased incidence of gastrointestinal adverse events compared to GLP-1 receptor agonists [8], co-prescribing these 2 medications is generally not recommended by major diabetes guidelines due to redundant mechanisms, increased gastrointestinal side effects, extra cost, and lack of added benefit [9]. 

Use of GLP-1 and GIP/GLP-1 receptor agonists has traditionally not been considered a risk factor for metformin-associated gastrointestinal adverse reactions, but based on the 4 cases we observed over the course of less than 12 months, we propose that there might be side-effect synergism when metformin and a GLP-1 or a GIP/GLP-1 receptor agonist are co-prescribed, especially knowing the similarity of their gastrointestinal adverse events, which might predispose susceptible individuals with type 2 diabetes to volume depletion, acute decline in kidney function, and ensuing unintentional metformin toxicity, including its most serious life-threatening manifestation, MALA. We summarized 4 cases at a single institution over 9 months of confirmed MALA in patients with type 2 diabetes mellitus co-prescribed metformin and semaglutide or tirzepatide (Table 1). All 4 patients were older, ages 67 – 69 years old, and overweight-to-obese with a body mass index of 25.9 – 40.4 kg/m^2^. They all had presumed normal kidney function prior to presentation and suffered from severe acute kidney failure requiring emergent renal replacement therapy. At presentation, the serum metformin level was markedly elevated, ranging from 8 to 31 µg/mL. The metformin therapeutic range is ~ 1 – 2 µg/mL, and MALA is known to develop at a metformin level exceeding 5 µg/mL [10]. Fortunately, all 4 patients recovered and were discharged from the hospital off dialysis. 

Semaglutide is recommended in patients with type 2 diabetes mellitus who have or are at risk for atherosclerotic cardiovascular disease or who have chronic kidney disease. The starting dose of the subcutaneous formulation of semaglutide is 0.25 mg/week, which is intended to reduce gastrointestinal symptoms, with up-titration up to a maximum dose of 2 mg/week to achieve glycemic goals. Similarly, tirzepatide may be preferred in patients with type 2 diabetes mellitus who might also benefit from weight loss due to the presence of weight-related comorbidities (such as symptomatic heart failure with preserved ejection fraction, or metabolic dysfunction-associated steatohepatitis). The initial subcutaneous dose of tirzepatide is 2.5 mg/week to minimize gastrointestinal symptoms, with up-titration to a maximum weekly dose of 15 mg. In a post-hoc analysis of the SURPASS-2 trial, tirzepatide improved therapeutic target attainment compared with semaglutide in patients with type 2 diabetes, including better glycemic control and weight loss [11]. Three of our patients were on a semaglutide dose of 1.0 – 2.0 mg/week while 1 was on a tirzepatide dose of 5 mg/week, and all 4 were on a maintenance metformin dose of 2,000 – 2,500 mg/day. In a network meta-analysis comparing the efficacy and safety of 9 GLP-1 receptor agonists and 1 GIP/GLP-1 dual receptor agonist (tirzepatide) as add-on to metformin in patients with type 2 diabetes, semaglutide (1.0 mg/week) and tirzepatide (15 mg/week) had a significantly higher rate of adverse events compared with the other GLP-1 receptor agonists [12]. We can only speculate as to whether our patients who were already on high-dose metformin (with 1 patient being on the maximum recommended dose) representing 80 – 100% of the total maximum daily dose of 2,500 mg (in the setting of preserved kidney function), when co-prescribed semaglutide or tirzepatide, became at risk for worsening gastrointestinal adverse reactions with the introduction or up-titration of these 2 medications, or had a heightened risk of acute kidney failure and metformin toxicity when they suffered from an acute gastrointestinal illness that likely predisposed them to volume depletion while continuing to take both metformin and a GLP-1 receptor agonist. In 2 of our 4 cases, there might have been other inciting events in addition to the ongoing use of a GLP-1 receptor agonist that might have led to MALA. In case 3, the patient had a recent influenza-like illness, leading to decreased oral intake. In case 4, the patient had a history of diverticulitis, which could have preceded the development of MALA although this could not be fully ascertained. 

In a case series of 6 patients with type 2 diabetes mellitus co-prescribed metformin and a GLP-1 receptor agonist, at baseline, all were taking metformin at a dose 2,000 – 2,500 mg/day (in divided doses before meals or otherwise on an empty stomach), with no gastrointestinal adverse reactions. Following the add-on of a GLP-1 receptor agonist, all patients began experiencing gastrointestinal adverse reactions, including nausea (n = 1), diarrhea (n = 3), or both (n = 2). These gastrointestinal adverse reactions resolved within 48 – 72 hours of stopping metformin and did not recur when metformin was resumed but taken immediately after eating. In 3 patients, the GLP-1 receptor agonist dose was increased to the maximum approved dosage without recurrence of gastrointestinal adverse reactions [13]. The authors concluded that treatment with a GLP-1 receptor agonist can unmask the gastrointestinal adverse reactions of metformin, and that post-prandial administration of metformin might allow some patients to tolerate a full dose of both medications, with potential for greater benefit in the treatment of type 2 diabetes mellitus. In a pharmacovigilance analysis that used the FDA Adverse Event Reporting System (FAERS) database to examine the real-world safety profile of tirzepatide, women experienced a greater risk of nausea compared to men, and older persons (age > 65 years old) exhibited a greater risk of abdominal discomfort, abdominal pain, constipation, diarrhea, dyspepsia, and flatulence compared to younger persons (18 – 65 years old) [14]. In this analysis, tirzepatide also showed a greater risk of gastrointestinal adverse events compared to metformin, and a similar gastrointestinal tolerability profile compared to GLP-1 receptor agonists. Our hypothesis-generating observations in the present case series require rigorous investigations of drug-drug interactions with GLP-1 receptor agonists (given their increased prescription rate) in an era of highly prevalent polypharmacy, particularly in older adults, and the need for rigorous post-marketing pharmacovigilance studies. One meta-analysis of older community-dwelling adults identified a drug-drug interaction pooled prevalence rate of 30.3% (using the Lexi-Interact tool) compared to a prevalence rate of 16.6% (using the 2015 American Geriatrics Society Beers criteria) [15]. 

When prescribing a GLP-1 receptor agonist or a GIP/GLP-1 dual receptor agonist, the current general guidance focuses on a dose reduction of insulin and/or insulin secretagogues (e.g., sulfonylureas and meglitinides) to avoid hypoglycemia and avoiding concomitant use with a DPP-4 inhibitor due to lack of additive glycemic benefit. There is currently no published guidance on how to manage metformin when a GLP-1 or a GIP/GLP-1 receptor agonist is co-prescribed. The authors recommend that prudence be exercised when co-prescribing these 2 classes of medications with close monitoring of kidney function and possibly reducing the dose of metformin, and there should be emphasis on sick day rule teaching, similar to what is now well established for patients prescribed a sodium-glucose cotransporter 2 inhibitor. 

## Authors’ contributions 

HH, MA, and KL drafted the manuscript. MCC, VSB, and BLJ were the physicians directly involved in the care of the patients. HH, MA, VSB, and BLJ revised the manuscript and contributed to the Discussion. HH, MA, and BLJ take responsibility that this case series has been reported honestly, accurately, and transparently, and accept accountability for the overall work by ensuring that questions about the accuracy or integrity of any portion of the work are appropriately investigated and resolved. 

## Funding 

This research received no funding. 

## Conflict of interest 

The authors declare no conflict of interest. 

**Table 1.** Characteristics, clinical course, and outcome of 4 patients with type 2 diabetes mellitus co-prescribed metformin and a GLP1 receptor agonist or GIP/GLP1 dual receptor agonist presenting with severe metformin-associated lactic acidosis.

**Case No.**	**Age**	**Gender**	**Clinical presentation**	**Weight (kg)/body mass index ** **(kg/m** ^2^ **)**	**Hemoglobin A1c**	**Metformin daily dose**	**GLP-1/GIP RA and weekly dose**	**On ACEI/ARB**	**Baseline serum creatinine, mg/dL**	**Initial serum creatinine, mg/dL**	**Initial lactic acid level, mmol/L**	**Initial serum bicarbonate, mmol/L**	**Initial serum anion gap, mmol/L**	Serum metformin level, mcg/mL*	**RRT requirement**	**RRT modality**	**RRT duration, hours**	**Serum creatinine at hospital discharge, mg/dL**
1	69	Female	Nausea, vomiting, and hypoglycemia	82.2/30.9	10.6%	2,000 mg	Semaglutide (1 mg)	Lisinopril	1.1	8.7	14.5	4	39	23	Yes	CVVHD	65	2.2
2	68	Male	Nausea and vomiting, hypoglycemia	120.9 (40.4)	8.3%	2,000 mg	Tirzepatide (5 mg)	Losartan	0.7	9.1	7.8	15	26	22	Yes	CVVHD	7	1.9**
3	67	Male	Chest and abdominal pain, vomiting, diarrhea, and hypoglycemia	90.1/26.9	8.8%	2,500 mg	Semaglutide (2.5 mg)	Lisinopril	1.1	8.2	20.2	3	40	31	Yes	CVVHD	72	1.4
4	67	Female	Nausea and vomiting	63.9/25.9	5.7%	2,000 mg	Semaglutide (2 mg)	Lisinopril	NA	5.3	14.3	9	40	8	Yes	IHD	4	3.0

GLP1-RA = glucagon-like peptide-1 receptor agonist; GIP = glucose-dependent insulinotropic polypeptide; RRT = renal replacement therapy; CVVHD = continuous veno-venous hemodialysis; IHD = intermittent hemodialysis; ACEI = angiotensin-converting enzyme inhibitors; ARB = angiotensin receptor blocker; NA = not available. *The serum metformin therapeutic range is ~ 1 – 2 µg/mL (reporting limit is 0.1 µg/mL), and metformin-associated lactic acidosis has been associated with metformin concentration exceeding 5 µg/mL; **10 weeks post hospital discharge.
